# Fluorescence microscopy reveals molecular localisation at line defects in nematic liquid crystals

**DOI:** 10.1038/srep36477

**Published:** 2016-11-04

**Authors:** Takuya Ohzono, Kaoru Katoh, Jun-ichi Fukuda

**Affiliations:** 1 Research Institute for Sustainable Chemistry, National Institute of Advanced Industrial Science and Technology (AIST), 1-1-1 Higashi, Tsukuba 305-8565, Japan; 2 Biomedical Research Institute, AIST, 1-1-1 Higashi, Tsukuba 305-8566, Japan; 3 Research Institute for Computational Design of Advanced Functional Materials, AIST, 1-1-1 Umezono, Tsukuba 305-8568, Japan

## Abstract

Topological defects easily form in liquid crystals (LCs) as a result of frustrations in spatially dependent anisotropic molecular ordering, and have been regarded as promising tools for facilitating manipulation of relatively large non-LC materials such as colloids. However, it remains unclear whether low-molecular-weight (LMW) impurities that do not aggregate or self-assemble in bulk LCs because of the dominance of entropy can localise at LC defects. Here, by fluorescence microscopy, we directly show the localisation of LMW molecules at the topological line defects of a nematic LC. It is theoretically explained that excess free energy density of nematic ordering at the defect core allows LMW solutes to accumulate at a non-negligible level overcoming the entropy leading to their uniform distributions. Our results demonstrate the usefulness of LC defects as a bottom-up field that enables micromanipulation of LMW molecules and realisation of transformable three-dimensional micro-architectures composed of versatile small functional molecules.

In thermal equilibrium, when entropy dominates the behaviour of solute molecules in a dilute solution, the concentration distribution of the solute molecules is spatially and temporally homogeneous, with thermal fluctuations satisfying the Boltzmann distribution. The entropic and the enthalpic contributions to the chemical potential of the solute molecules are balanced and spatially uniform. However, a non-uniform field applied to the solution alters the enthalpic part of the chemical potential and gives rise to non-uniform concentration distributions. Well-known examples include the electrical double layer electrostatically-induced by a charged boundary for an electrolyte solution^1^. Appropriate choice of the field to be applied to a solution thus provides a convenient means of manipulating solute molecules in a solution.

The positional and/or orientational order of a liquid crystal (LC) can serve as such a non-uniform field^2^,^3^,^4^. The orientational field of a nematic liquid crystal (NLC), the simplest non-trivial phase of a LC with long-range orientational order and no positional order, is commonly characterized by a unit vector ***n*** known as the director. How strongly the LC molecules are orientationally ordered introduces another order parameter denoted by a scalar *S*. It is well known that most of non-LC molecules that can dissolve into NLCs decrease *S*, and therefore such non-LC molecules would favour a region with smaller *S*. Indeed, Samitsu *et al*.^5^ demonstrated, by designing a system made of a photo-responsive LC, that a region of smaller *S* induced by light irradiation attracts small polymeric impurities, which results in their non-uniform distribution. Thus, the control of the profile of *S* can provide a means of manipulating non-LC components.

Although in usual NLCs *S* is regarded as constant except close to the nematic-isotropic transition, *S* adopts a smaller value at the core of topological defects, or singularities in ***n***^6^,^7^. Topological defects in NLCs have been known to trap or interact with colloidal and polymer particles^8^,^9^,^10^,^11^,^12^,^13^,^14^ and therefore offer an intriguing platform for micro-manipulation of small objects in fluid phases, because the position and shape of defects can be externally controlled and modulated, for example, by boundary conditions, an electric field and light. The objects whose manipulation by defects has been reported so far were much larger in size than the NLC molecules, and were attracted by the defects even if the distance between the object and the defect was much larger than the object size. Since the distribution of smaller *S* due to defects is highly localised (whose characteristic width is known as the nematic coherence length and on the order of 10 nm), such large objects do not interact with the field of *S*, but with that of ***n*** that exhibits long-range distortions by the defects. However, when the possibility of manipulating low-molecular weight (LMW) molecules by LC defects is concerned, ***n*** does not seem to play a role because it is regarded as uniform at the molecular scale. Therefore, non-uniform profile of *S* should be exploited and a question then arises as to whether that induced by a topological defect is sufficient to drive the non-uniform concentration distribution of LMW molecules. The effect of *S* must overcome the contribution of entropy leading to their uniform distributions. Very recently, Wang *et al*. reported^15^,^16^ that a class of surfactant molecules assemble specifically at the defect core. However, they also showed that the distribution of surfactant monomers was uniform (see Fig. 2b,e of ref. 15). Therefore it is not yet clear whether non-uniform concentration distribution of LMW molecules can be induced by defects.

## Results

**Figure 1:**
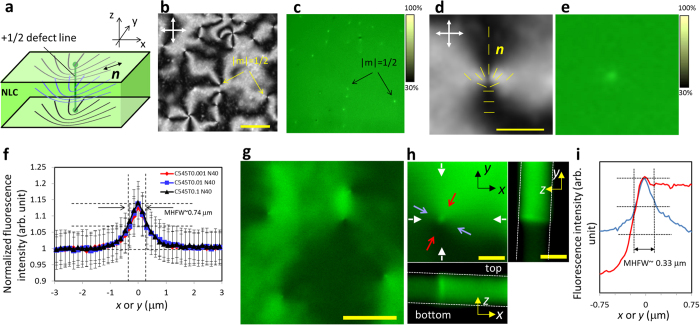
Gleaming defects in a NLC doped with LMW fluorescent probe in a thin planar cell, System-(i). (**a**) Schematic for the NLC cell accommodating a disclination line with *m* = +1/2, shown by the green line. The top and bottom surfaces impose isotropic planar alignment to the NLC. The curved solid lines indicate the orientation of ***n***, basically lying in the *xy* plane in System-(i). Representative (**b**) POM and (**c**) FOM images, showing many defects with *m* = ±1/2 appearing as gleaming points in the FOM image obtained for the sample at *c* = 0.1 wt%. (Scale bar: 20 μm.) Representative magnified (**d**) POM and (**e**) FOM images of a defect with *m* = +1/2. The contrast of FOM images was enhanced from that of original images for visibility; the upper 70% of the original dynamic range of fluorescence detection was assigned to the attached colour chart. (Scale bar: 5 μm) (**f**) Fluorescence intensity profiles averaged over N = 40 gleaming defects obtained at different concentrations of C545T, 0.001, 0.01, and 0.1 wt%. Each fluorescence profile was normalized by intensity obtained at a distance of ~3 μm from the peak. Representative CLS-FOM image (**g**) (Scale bar: 5 μm) and that of a single line defect with cross sectional images (**h**) (Scale bar: 1 μm) obtained for the sample at *c* = 0.1 wt% (see Supplementary Fig. 3 for other images). Away from the gleaming defects, weak distribution of the fluorescence intensity can be seen that corresponds to the in-*xy*-plane nematic director distribution, (*n*_*x*_, *n*_*y*_), caused by weak linear polarization of the excitation laser in the *y* direction. The positions of *xz* and *yz* cross sections are indicated by white arrows. (**i**) Fluorescence intensity profiles of the gleaming defect shown in (**h**) along two lines indicated by blue and red arrows. The intensity peak at the defect is confirmed also in the red profile, which is asymmetric due to the polarization of the excitation laser.

Here, line defects formed in a NLC (pentylcyanobiphenyl, 5CB) doped with a fluorescent LMW probe (coumarin 545T, C545T, see Supplementary Fig. 1 for fluorescence spectrum) were observed using a polarizing optical microscope (POM) in transmission mode to identify the locations of defects. Moreover we used two fluorescence optical microscopes (FOMs); a conventional FOM and a confocal laser scanning (CLS) FOM^17^,^18^,^19^,^20^. Molecular localisation at the defect was characterized by the fluorescence images (Fig. 1). Line defects with a winding number *m* of ±1/2 were prepared in two systems (see Methods); System-(i): a thin planar cell without preferential surface alignment orientation, in which line defects with *m* = ±1/2 run between the top and the bottom boundary substrates (Fig. 1a), and System-(ii): a thin NLC filament formed on an anisotropic open capillary^12^,^21^ with a sinusoidal cross sectional shape imposing semi-hybrid alignment, in which a zigzag line defect with *m* = +1/2 runs along the capillary^12^,^22^. In both cases defects are clearly gleaming, evidencing the molecular localisation there.

**Figure 2:**
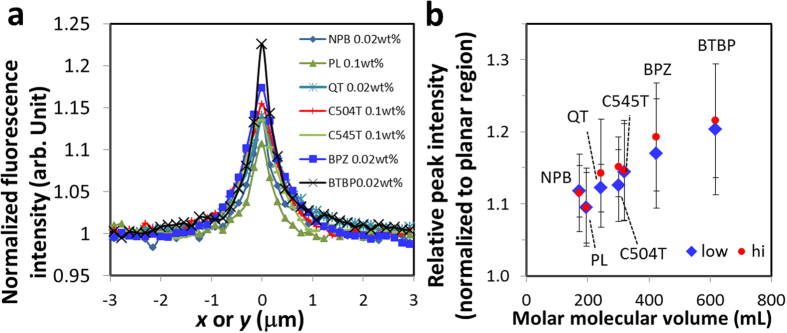
Analysis of gleaming defects obtained for various LMW fluorescent probes in 5CB. (**a**) Fluorescent intensity profiles averaged over N = 40 gleaming defects, obtained for different probes; NPB (0.02 wt%), PL (0.1 wt%), QT (0.1 wt%), C504T (0.1 wt%), C545T (0.1 wt%), BPZ (0.02 wt%), and BTBP (0.1 wt%). Each fluorescence profile was normalized by intensity obtained at a distance of ~3 μm from the peak. The error for each data point was approximately ±5~7% (N = 40), which was not shown in the plot for visibility. (**b**) The averaged peak values (N = 40) were plotted with respect to the molar molecular volume in their solid states, suggesting a positive correlation between them. The data include the values obtained for two different concentrations indicated by ‘low’ and ‘hi’; NPB (0.01, 0.02 wt%), PL (0.01, 0.1 wt%), QT (0.01, 0.1 wt%), C504T (0.01, 0.1 wt%), C545T (0.01, 0.1 wt%), BPZ (0.01, 0.02 wt%), and BTBP (0.01, 0.1 wt%).

In System-(i), POM images exhibit a typical Schlieren pattern with defects as points, each accompanied by two dark brushes that rotate on sample rotation depending on the sign of m (Fig. 1b,d). The point-like appearance of the defects indicates that each point corresponds to a line defect running almost parallel to the optical axis of the microscopy observation, *z*, throughout the cell thickness (2 ± 1 μm in our experiments). In FOM images (Fig. 1c,e), bright spots appeared at exactly the same places where the defects are located in the POM images. The half maximum full-width (HMFW) of the intensity profiles were approximately 0.7 μm, independent of the C545T concentration in the range we studied, 0.001~0.1 wt% (Fig. 1f). This independence was also found using other LMW fluorescent probes (Fig. 2).

One can obtain the information on the profile of the director ***n*** from the fluorescence intensity profile *I*(*x*, *y*) of FOM. It is well known that most of the fluorescent molecular probes including the one we used in our experiments, C545T, align in NLCs along ***n*** ( = [*n*_*x*_, *n*_*y*_, *n*_*z*_] and |***n***| = 1) at a certain degree^23^. When the incident light is polarized along a certain direction ***a*** in the FOM, the excitation efficiency is proportional to (***n*** · ***a***)^2^, and therefore the fluorescent intensity is maximum when ***n*** is along ***a*** (see Supplementary Fig. 2A,B). In the present FOM, the excitation light is unpolarized and propagates along the optical axis *z* on average, and the excitation efficiency is now proportional to 〈*n*_*x*_^2^〉 + 〈*n*_*y*_^2^〉 = 1 − 〈*n*_*z*_^2^〉, thus dependent only on *n*_z_ (Here 〈〉 denotes the average in the *z* direction over the NLC thickness). The collected fluorescence intensity through the identical optical axis *I* is therefore proportional to (1 − 〈*n*_*z*_^2^〉)^2^ (refs 19, 20).

In System-(i) with a thin planar cell, *n*_*z*_ basically equals zero, which would yield uniform *I* if the C545T concentration were uniform. Moreover, as the C545T molecules may orient out of the *xy* plane at the defects because of weaker order of the host LC there^5^,^6^, the fluorescence efficiency at the defects would be reduced, leading to darker appearance of the defects. Therefore, the gleaming defects indeed indicate higher C545T concentration there and should not be attributed to an artefact caused by the dichroic property. Moreover, it was confirmed that the degree of the dichroic properties measured for other fluorescent probes showed no correlation with their averaged relative peak intensities of the gleaming defects (Fig. 2b, Supplementary Fig. 2C). The result again supports our interpretation.

Furthermore, gleaming defect lines with the HMFW being approximately 0.33 μm were also resolved using CLS-FOM with a careful optical setup to reduce the spherical aberration (Fig. 1g–i, Supplementary Fig. 3A,B). As the excitation laser was linearly polarized in our CLS-FOM (see Methods), nonuniform distribution of the fluorescence intensity corresponding to that of ***n*** around the defect was found. Nevertheless, the fluorescence intensity at defects was again higher than that away from the defects (Fig. 1h,i). Strong spatial variation of the dielectric tensor at the defects might cause optical artefacts, such as polarization guiding and other birefringent effects. However, defect lines with slightly curved and/or oblique shape in random directions, especially at larger thickness, were clearly resolved across the full thickness of the cell (see also Supplementary Fig. 3B,C). The fluorescent images did not show any systematic distortion in the *z* direction resulting from the defects. The results indicate that the optical paths of the excitation laser and the collected fluorescence are hardly distorted even around the defects, and thus, an exact three-dimensional fluorescent intensity map is obtained within the present optical resolution. Ultimately, the CLS-FOM observation also supports the molecular localisation at the defects.

**Figure 3:**
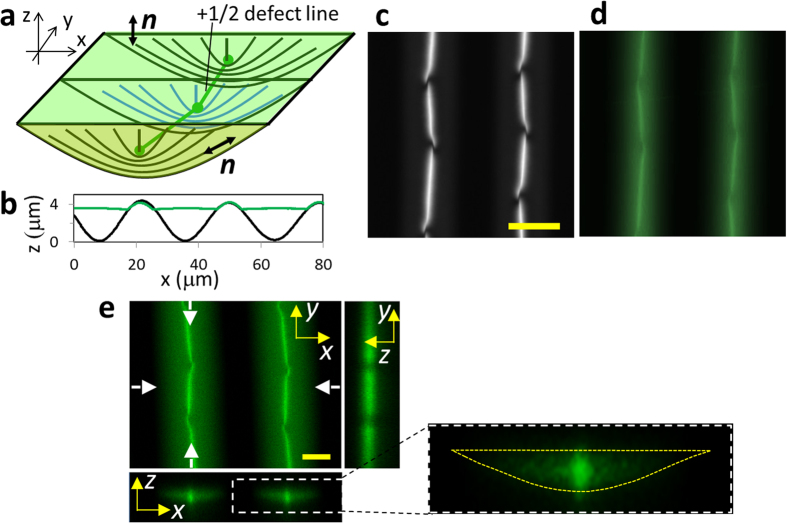
Gleaming defects in a NLC doped with LMW fluorescent probe confined within open capillary grooves, System-(ii). (**a**) Schematic for the filament of a NLC formed on an open capillary groove accommodating a zigzag disclination line with *m* = +1/2 shown by green lines (see ref. 12 for the detailed alignment structure). The top air interface imposes homeotropic (normal) alignment to the NLC. The bottom curved surface imposes anisotropic planar alignment lying in the *xz* plane along the sinusoidal cross section. Under this semi-hybrid alignment condition and the sign of the curvature of the groove, disclination lines with *m* = +1/2 stably form. The curved solid lines indicate the orientation of ***n***. Twist deformations of ***n*** formed under the defect lines are not shown for simplicity. (**b**) Surface profiles of the open capillary with (green line) and without (black line) the NLC filaments. Representative (**c**) POM and (**d**) FOM images showing zigzag line defects with *m* = +1/2, appearing as gleaming zigzag lines in the FOM image. (Scale bar: 20 μm.) (**e**) A representative CLS-FOM image and the cross sectional images in the *xz* plane, in which the region occupied by the NLC in the *xz* plane is indicated by yellow broken lines in the magnified image (right). It is indicated that the gleaming line runs halfway between the top air surface and the bottom surface of the NLC filament, that is, the line defect gleams. The positions of *xz* and *yz* cross sections are indicated by white arrows. (Scale bar: 10 μm).

In System-(ii) (Fig. 3a,c), gradual change of *n*_*z*_ along the *z* direction, together with the curved bottom boundary and resulting thickness variation depending on the position, complicates the interpretation of FOM images. Nevertheless, the gleaming zigzag defect line with *m* = +1/2 was resolved using the FOM (Fig. 3d and see Supplementary Fig. 4 for gleaming zigzag defects with *m* = −1/2). Although the resolution of the CLS-FOM images (Fig. 3e) is lower than that of System-(i) due to the optical setup, it is indicated that the gleaming line runs halfway between the top air surface and the bottom substrate of the NLC filament, that is, the line defect gleams.

**Figure 4:**
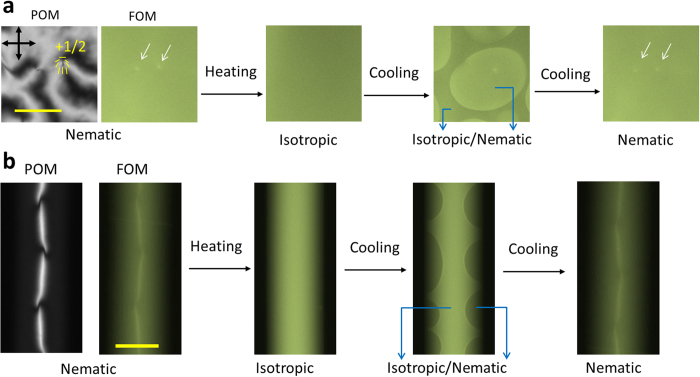
Recovery of gleaming defects through phase transitions. The initial states at room temperature (in the nematic state) observed by POM and FOM are shown for both System-(i) (**a**) and System-(ii) (**b**). As soon as the liquid crystal becomes isotropic on elevating temperature (~36 °C), the gleaming defects (white arrows) disappear and homogeneous fluorescence intensity is observed. The gleaming defects reappear soon after the transition to the nematic state. In System-(i), the defects reappear at almost the same positions, which is probably due to the surface memory effect^29^,^30^,^31^. The average intensity in the isotropic state is lower than that in the nematic one, because of the random distribution of the orientation of the transition moment of fluorescent molecules ***P***, in contrast to its distribution in the *xy* plane in the nematic state (see main text). In System-(ii), the average intensity in the isotropic state is higher than that in the nematic state. This is due to the hybrid alignment formed in System-(ii)^12^,^22^,^24^,^32^ in the nematic state; near the liquid crystal-air boundary, ***P*** is almost parallel to the optical axis and therefore the fluorescent molecules are hardly excited. (Scale bars: 20 μm).

To confirm that the state of the molecular localisation at defects was in thermal equilibrium, FOM observations were conducted through a cycle of increasing and decreasing temperature (Fig. 4a,b). The gleaming points and lines in the N state readily disappeared in the isotropic state and reappeared as soon as the system was quenched to the N state. Moreover, gleaming defects were observed using other various LMW fluorescent probes (Fig. 2 and Supplementary Table 1), demonstrating the generality of molecular localisation at the line defects of NLCs.

## Discussion

The determination of accurate distribution of the guest fluorescent molecules from experimental data is prohibitively difficult because of the limitation of resolution in optical microscopy. Here we exploit a simple theoretical model to demonstrate that the localisation of guest LMW molecules at the defect core of the host liquid crystal is indeed possible even when entropy dominates their distribution. We denote the volume fraction of the guest molecules at the defect core by 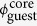 and that in the bulk by 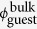. Then we have (see Methods)


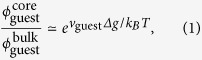


where *v*_guest_ is the volume occupied by one guest molecule, *Δg* is the excess free energy density of the defect core of the host liquid crystal, *k*_*B*_ is the Boltzmann constant, and *T* is the temperature. Since *k*_*B*_*T* = 4.1 × 10^−21^ J, and a rough estimate gives *v*_guest_ ≃ 6 × 10^−28^ m^3^ and *Δg* ≃ 10^6^ Jm^−3^ (see Methods), we obtain 
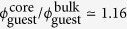
. This estimation indicates that the excess free energy of the defect indeed drives the guest molecules towards the defect core, overcoming entropy that would yield a uniform distribution. Moreover, this value of 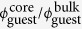 is close to the intensity ratio, although one cannot safely identify the intensity distribution with that of the guest fluorescent molecules because of the limitation of resolution in microscopy, inherent fluctuations in the NLC around the defect^24^, and a possible effect of concentration quenching. Nevertheless, the present crude theoretical argument is consistent with the experimental findings that the intensity ratio is independent of the concentration (Fig. 1f) and positively correlated with the molecular volume (Fig. 2b).

In conclusion, we have observed the localisation of LMW fluorescent probes at the line defects in a NLC. This is also supported by a theoretical model considering the coupling between the dopant concentration and higher free energy density of the host NLC at the defect core. We believe that even in the system reported by Wang *et al*.^15^,^16^ gleaming defects due to monomeric fluorescent surfactants will be detectable if the optical setup is carefully optimized. Such an observation may be useful to understand the self-assembling process at the defect in more details. However, for more quantitative analysis of the molecular localisation, a careful study of the effect of concentration quenching and a detailed understanding of the image formation mechanism in optical microscopy will be required, although the latter problem regarding an anisotropic medium containing sub-resolution-scale spatial inhomogeneity is highly challenging theoretically. Nevertheless, our demonstration of the localisation of low-molecular-weight molecules at liquid crystal defects will help to design complex nanostructures utilizing defects such as those in LC blue phases^25^ as a template, and will be a step towards the realisation of defect-based micromanipulation and transformable three-dimensional architectures^8^,^9^,^10^,^11^,^12^,^13^,^14^,^15^ composed of versatile small functional molecules. Moreover, our concept of micromanipulation of LMW molecules by topological defects is applicable to general LCs other than a nematic LC in our study, and therefore offers a means to improve the functionality of a wide variety of LC-based advanced materials, e.g., LC elastomers, colloid-LC composites, and composites with lamellar structures, by introducing functional molecules into the structures.

## Methods

### Materials

Nematic (N) liquid crystal (LC) 4-cyano-4′-pentylbiphenyl (5CB) was purchased from Wako Chemical. The fluorescent probe coumarin 545T (C545T) was purchased from Tokyo Chemical Industry. Poly-amic acid (Poly(pyromellitic dianhydride-co-4,4′-oxydianiline) for a precursor solution of polyimide coating for planar alignment and *N*-methylpyroridone (NMP) for the solvent were obtained from Sigma-Aldrich. The silicone elastomer kit, Sylgard 184 (polydimethylsiloxane, PDMS), for the substrate of the wrinkled sinusoidal open capillary was obtained from Toray-Dow-corning. The silicon substrate (n-type, 4 inch) was obtained from Fruuchi Chemical. The polyimide spacer (Kapton^®^, thickness ~12.5 μm) was obtained from Nilaco. Cover glass slides (No. 1S, 22 × 22 mm^2^) were obtained from Matsunami Glass Industry.

Regarding other LMW fluorescent probes, 4-Nitro-7-piperazino-2,1,3-benzoxadiazole (NPB), α-quaterthiophene (QT), fluorescein, coumarin 504T (C504T), Bis[2-(2-benzothiazolyl)phenolato]zinc(II) (BPZ), were obtained from Tokyo Chemical Industry, perylene (PL) was obtained from Nacalai tesque, N,N’-Bis(2,5-di-tert-butylphenyl)-3,4,9,10-perylenedicarboximide, (BTBP) and rubrene were obtained from Sigma-Aldrich, and 25-[N-[(7-nitro-2-1,3-benzoxadiazol-4-yl)methyl]amino]-27-norcholesterol (NBD-cholesterol) was obtained from Avanti Polar Lipids.

### Preparation of planar LC cells

We adopted a wedge cell with planar alignment without preferential direction to observe a very thin region at the thickness of roughly less than 3 μm. A spin-coater (MSA-150, Mikasa) was used at 5,000 rpm for 60 s to coat cover glass slides with a poly-amic acid solution (1 wt%). The coated slides were then heated on a hot plate at 70 °C for 2 min to evaporate the solvent, and subsequently placed in an oven at 180 °C for 3 h to induce the transformation of poly(amic acid) to polyimide through the dehydration reaction. Then, two slides were assembled to a wedge cell using a Kapton^®^ spacer placed at one side of the cell with a small amount of adhesive (liquid gasket1212, Threebond). The slides were held together using clips during curing the adhesive. Although the wedge angle was ideally ~0.036 deg, the actual cell showed spatial fluctuation of the wedge angle (and the cell gap), probably due to the bending of the cover glass and inhomogeneity in the thickness of the polyimide layer. The NLC, 5CB, doped with C545T at the concentration, *c*, of 0.001, 0.01, or 0.1 wt% was injected into the wedge cell at an elevated temperature (40~45 °C) via capillary action. The sample was cooled to room temperature (22 ± 2 °C) and used for microscopic observations of System-(i). Samples doped with other LMW fluorescent probes were prepared by the same procedure.

### Preparation of NLC filaments confined within wrinkled grooves

We prepared wrinkles with aligned grooves, an array of open capillaries, by the following procedure identical to that reported previously in refs 12, 21. The PDMS elastomer (1:10 [by weight] = [curing agent]:[polymer]) was cured on a smooth Si wafer. The smooth surface of the PDMS substrate (12 × 12 × 6 mm^3^) was weakly treated by an Ar plasma (SEDE-P, Meiwa Forsis) to enhance the wetting of NMP. Soon after an NMP solution of poly-amic acid solution (7.5 wt%) was coated on the activated PDMS surface using the spin-coater at 5,000 rpm for 60 s, the substrate was uniaxially compressed with the strain of approximately −0.05 (−5%). And the sample was heated under the compression at 180 °C for 3 h for imidization. During the temperature increase in this heating process, the PDMS sample dilated to the direction perpendicular to the compression due to thermal expansion. This expansion during the imidization plausibly aligned the main chains of the polymer anisotropically, which imposed the uniaxial alignment of 5CB as described below. After cooling down to 25 °C and the release of the compression, aligned wrinkle grooves were formed with the easy axis of the liquid crystal alignment perpendicular to the groove direction. The periodicity of the groove *λ* was 27~29 μm. The aspect ratio of the wrinkles *R* ≡ *D*/λ, where *D* is the groove depth, was 0.145 ± 0.01, which was characterized using a laser microscope (VK-9710, KEYENCE).

We prepared NLC filaments on the wrinkle grooves in the following way, and refer to Supplementary Figure 1 of ref. 12 for details. A bendable polyimide (Kapton^®^) film was fixed to an adjustable bar at one end and was hung over the surface of the wrinkles. After a small droplet (1~5 μL) of the NLC, 5CB doped with C545T was placed on wrinkles at an elevated temperature (40~45 °C), the polyimide film was bent towards the droplet and brought close to the wrinkles. Consequently, the film stuck to the wrinkles due to the attractive capillary force from the bridged liquid, making a straight triple line of solid (wrinkle substrate), liquid and air. To fill the wrinkle grooves with the liquid, the polyimide film was moved along the groove direction. The NLC was successfully introduced into only the grooves, and a certain portion of the crest part was still exposed to the air. The surface profile of wrinkles with liquid filaments was characterized using a laser microscope. Zigzag defect lines with *m* = +1/2 were formed along groove (Supplementary Fig. 5). Occasionally, the top of the crest part was also covered by the NLC, in which we had a chance to observe zigzag defect lines with *m* = −1/2 formed along the crests.

### Optical microscopy

We observed the LC alignment using a transmitted polarizing microscope (POM) (BX-51P, Olympus) under crossed-Nicol conditions. To differentiate small optical rotations visually by the colour difference, a 530 nm sensitive tint plate (1 λ, U-TP530, Olympus) was also used.

To observe the fluorescent images, we used a conventional fluorescence microscope (FOM), where the light source was mounted above the sample and the excitation light passed through the microscope objective lens on its way toward the sample. A Xe lamp (75 W) was used as the light source. To detect the emission from C545T and other probes (NPB, QT, fluorescein, C504T, NBD-cholesterol, BTBP) emitting green fluorescence, we used a fluorescence filter set (U-MWIB-3, Olympus) comprising an excitation filter that transmitted light with wavelengths between 460 nm and 495 nm and an emission filter that transmitted light with wavelengths of >510 nm. For PL and BPZ, FOM images were acquired with another filter set (U-MWU2, Olympus); excitation 335~385 nm and emission >420 nm (blue fluorescence). A third filter set (U-MWIG3, Olympus), excitation 530 nm~550 nm and emission >575 nm, was used for red fluorescence from rubrene. The images were collected at the pixel size of 0.154 μm/pixel using a Nikon DS-Qi1-Mc CCD (charge-coupled device) camera connected to a computer and controlled through imaging software (NIS-Elements, Nikon). An objective lens with a numerical aperture (NA) of 0.95 (UPLSAPO40 × 2, Olympus) was mainly used. For measurements of dichroic properties, an objective lens with the numerical aperture of 0.75 (MPLFLN 50 × BDP, Olympus) was used.

In System-(i), we could find a region having a thickness of approximately 2 μm near the edge opposite to the spacer. By the POM under the crossed-Nicol condition, we observed a region showing the yellow colour (Supplementary Fig. 6). This region was in the first order colour range of the Michel-Levy interference chart [see, e.g., Olympus Microscopy Resource Centre homepage], and its thickness was estimated to be 2 ± 1 μm, since the birefringence of 5CB is ~0.18 at 23 °C^26^. For the analysis of the fluorescence intensity of the gleaming defect, the focus was carefully adjusted to give tack-sharp peaks. To reduce the errors from noise, the cross sections of randomly chosen fluorescence intensity distributions around defects (N = 40) were averaged after the background signal was subtracted. Each fluorescence profile was normalized by intensity obtained at a distance of ~3 μm from the peak.

A confocal laser scanning (CLS) FOM (A1^+^ system, Nikon) was used to obtain the three-dimensional fluorescence images (Figs 1g,h and 3e, Supplementary Fig. 3). An optically pumped semiconductor laser (LU-N4 Laser Unit, Nikon, equipped with Sapphire 488, Coherent Inc.) was used to excite the fluorescent molecules at 488 nm and the emitted light between 525 and 595 nm was collected. An objective lens with a NA of 1.45 (PlanApoTIRF100×, oil, Nikon), and one with NA of 0.75 (PlanApo20×, dry, Nikon) were used for the observations of System-(i) and System-(ii), respectively. The CLS-FOM images were acquired at the voxel size of typically 60(*x*) × 60(*y*) × 60(*z*) nm^3^ or 30(*x*) × 30(*y*) × 60(*z*) nm^3^. The excitation laser was weakly linearly polarized in *y* direction, which could cause the contrast of fluorescent intensity of the sample of System-(i) depending on the in-plane distribution of the NLC director over the observation region. The polarization of the excitation laser in the *y* direction could be confirmed using a planar cell with unidirectional alignment direction (KSRP-02/A111P1NSS05, EHC; cell gap of 2 ± 0.5 μm) filled with 5CB doped with C545T at 0.1 wt%; the dichroic ratio, that is, the fluorescence intensity ratio between the maximum (*y* axis) and minimum (*x* axis), was 2.4 ± 0.5.

The actual cell gap at the observation point was determined as the thickness of the fluorescent region in the *z* direction obtained using CLS-FOM.

The optical observations described above were carried out at room temperature (22 ± 2 °C). To investigate the effect of the phase transition between the nematic and the isotropic states, optical measurements were conducted on a hot plate with a temperature control (ThermoPlate, Tokai Hit). The temperature was changed at the rate of ~2 K/min.

### Theoretical argument

We consider a system in which guest molecules of local volume fraction *ϕ*_guest_(**r**) are distributed in the host LC containing a topological line defect (disclination). The Flory-type free energy of mixing reads


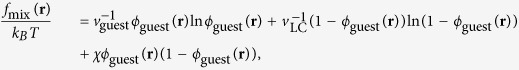


where *v*_guest_ and *v*_LC_ are the volume occupied by one guest molecule and by one LC molecule, respectively. From the incompressibility condition, the local volume fraction of the LC is 1 − *ϕ*_guest_(**r**). *χ* is the parameter characterizing the mixing enthalpy.

We assume that the guest molecules, because of their small amount, do not distort the orientational order of the host LC, and let *g*(**r**) denote the free energy density due to the orientational order of the (pure) LC. The free energy of the host LC is then written as *f*_LC_(**r**) = (1 − *ϕ*_guest_(**r**))*g*(**r**).

The total free energy of the system is given by 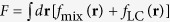, and the distribution of *ϕ*_guest_ is determined by


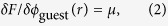


where *μ* is the chemical potential independent of **r**. We denote *g*(**r**) at the defect core by *g*_core_ and that in the bulk by *g*_bulk_. Then, eq. (2) yields


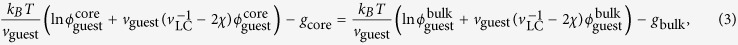


where we have dropped terms second order or higher in *ϕ*_guest_ because in our experiments *ϕ*_guest_ ≪ 1 (at most 10^−3^). We will show later that the contribution of the term 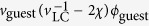 is much smaller that that of the other terms, and therefore we have


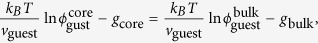


leading to eq. (1) because *Δg* = *g*_core_ − *g*_bulk_.

The volume *v*_guest_ can be roughly estimated by the molecular weight and the mass density. The molecular weight of C545T^27^ is *M* = 430.57, and its mass density is *ρ* ≃ 1.3 × 10^3^ kg/m^3^. Therefore, *v*_guest_ ≃ 10^−3^ *M*/*ρN*_*A*_ ≃ 6 × 10^−28^ m^3^ with *N*_*A*_ being the Avogadro constant (This is likely to be an underestimate because the above *ρ* is that of the crystal). The estimation of *Δg* is not trivial, and the common strategy is to rely on the Landau-de Gennes theory describing the orientational order of a nematic LC by a second-rank tensor *Q*_*ij*_, with *i*, *j* = *x*, *y*, *z*. The free energy density of the LC in terms of *Q*_*ij*_ is *f*_LCbulk_ + *f*_LCgrad_, where


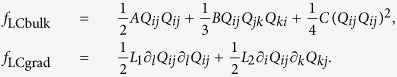


Here summations over repeated indices are implied, and *A*, *B*, *C*, *L*_1_ and *L*_2_ are material parameters (particularly, the temperature dependence of *A* accounts for the transition between the nematic state and the isotropic state). Although the accurate estimation of *Δg* requires the calculation of *Q*_*ij*_(**r**) that minimizes the free energy functional 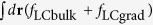, *Δg* is roughly estimated to be *B*^4^/*C*^3^ times a numerical factor of the order of unity. Since *B* and *C* are of the order of 10^6^ Jm^−3^, we have *Δg* ≃ 10^6^ Jm^−3^ (refs 6, 28).

Finally, we estimate the contribution of the term 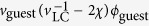 in eq. (3). Since we consider a system that does not phase separate spontaneously without topological defects of the host liquid crystal, let us consider the condition of this absence of phase separation. Whether the system phase separate or not is dictated by 
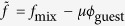
, where the chemical potential *μ* is determined so that there exist *ϕ*_a_ and *ϕ*_b_ (*ϕ*_a_  ≨ *ϕ*_b_) that satisfy 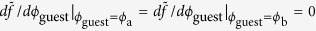 and 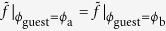 (if no set of (*ϕ*_a_, *ϕ*_b_, *μ*) satisfies these equations, phase separation does not occur whatsoever). In the simple case of *v*_LC_ = *v*_guest_, we obtain *μ* = 0 and 
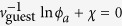
 (Here *ϕ*_*a*_ ≪ 1 has been assumed, and higher order terms in *ϕ*_*a*_ have been dropped). In our experiments, no phase separation without topological defects occurs at *ϕ*_guest_ = 10^−4^ − 10^−3^ < *ϕ*_a_, and therefore 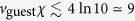. We can therefore safely conclude that 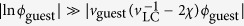 since *ϕ*_guest_ < 10^−3^. We also note, a posteriori (
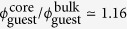
 leads to 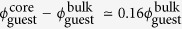), that, for *ϕ*_guest_ = 10^−3^, 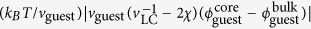 is of the order of 10^4^ J m^−3^, much smaller than *Δg* ≃ 10^6^ J m^−3^.

## Additional Information

**How to cite this article**: Ohzono, T. *et al*. Fluorescence microscopy reveals molecular localisation at line defects in nematic liquid crystals. *Sci. Rep.* **6**, 36477; doi: 10.1038/srep36477 (2016).

**Publisher’s note:** Springer Nature remains neutral with regard to jurisdictional claims in published maps and institutional affiliations.

## Supplementary Material

Supplementary Information
