# Supplementary material for: Fluorescence microscopy reveals molecular localisation at line defects in nematic liquid crystals
Source: Sci Rep. 2016 Nov 4;6:36477. doi: 10.1038/srep36477 (PMC5095605; doi:10.1038/srep36477)
Supplement: Supplementary Information [file srep36477-s1.pdf]

Supplementary Information

Fluorescence microscopy reveals molecular localisation at line defects in nematic liquid crystals

by Takuya Ohzono, Kaoru Katoh & Jun-ichi Fukuda

Supplementary Figures 1-6

Supplementary Table 1

Supplementary Figure 1| Fluorescence spectrum of C545T in 5CB. The fluorescence spectrum of C545T in 5CB at concentration of 0.1wt% (excitation at 475 nm) was measured using a spectrophotometer (V-630, Jasco). The LC was injected into a planar cell with unidirectional alignment treatment (KSRP-02/A111P1NSS05, EHC; cell gap of 2 ± 0.5 μm) at an elevated temperature of 40℃ and the sample was cooled to room temperature (22 ± 2℃) for measurement. This cell showed homogeneous planar alignment and no LC defect. The cell was placed on the sample holder of the spectrophotometer with the surface inclined by 45 degrees to avoid detection of the excited light and to collect only the fluorescence emission.

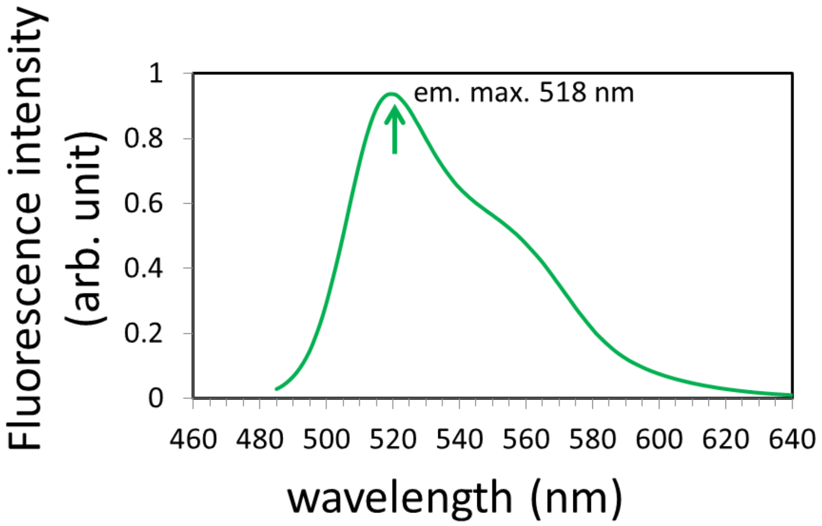

Supplementary Figure 2| Dichroic properties of C545T and other LMW fluorescent probes in 5CB. (A) FOM images of C545T (0.1wt% in 5CB) with a polarizer (grayscale). We evaluated the cell identical to that used for fluorescence spectrum measurement. The fluorescence intensity was measured using the FOM with a polarizer, through which both the incident excitation light and the emitted fluorescence passed. The average intensities at different angles, ϕ, between the axis of the polarizer and the nematic director of the cell were measured. The maximum and minimum intensities were obtained at ϕ = 0 deg and ±90 deg, respectively, indicating that the transition moment of C545T was aligned to the nematic director, ***n***. (B) The fluorescence intensities at different ϕ obtained for C545T and other LMW fluorescent probes in 5CB; NPB (0.02wt%), PL (0.1wt%), QT(0.1wt%), C504T (0.1wt%), BPZ (0.02wt%), and BTBP (0.1wt%). (C) Plot of the dichroic ratio $D = I_{max}/I_{min}$ vs. molecular volume per mol in their solid states, where I_{max} and I_{min} are the fluorescence intensities for each fluorescent probe shown in the plot (B). Comparing the results with those shown in Fig. 2b, it is clear that the degree of the dichroic property shows no correlation with the averaged relative peak intensities of gleaming defects.

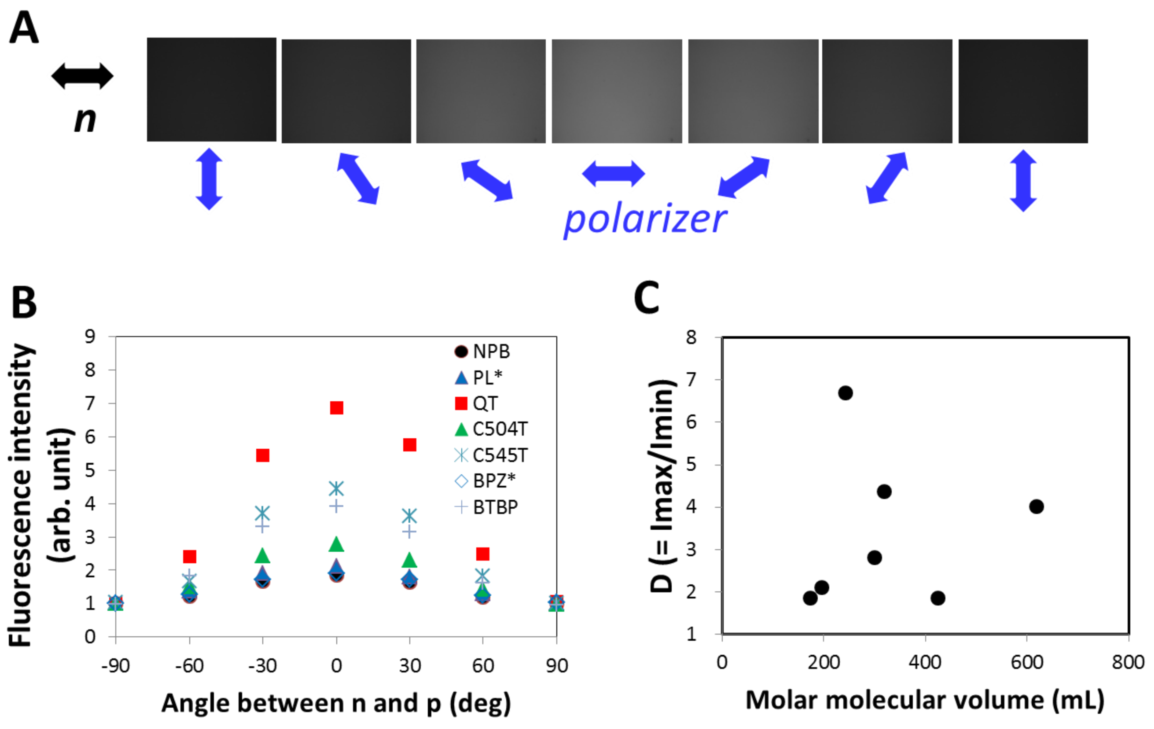

Supplementary Figure 3| Representative CLS-FOM images of line defects in system-(i). (A) Bird's eye view of the fluorescence intensity shown in Fig. 1h. The gleaming line defect is indicated by an arrow. Representative CLS-FOM cross-sectional images of line defects obtained for the sample doped with C545T (c = 0.1wt%) in (B) thin (thickness of ~1.4 μm) (Scale bars: 1 μm) and (C) thick (~7 μm) regions (Scale bars: 2 μm).

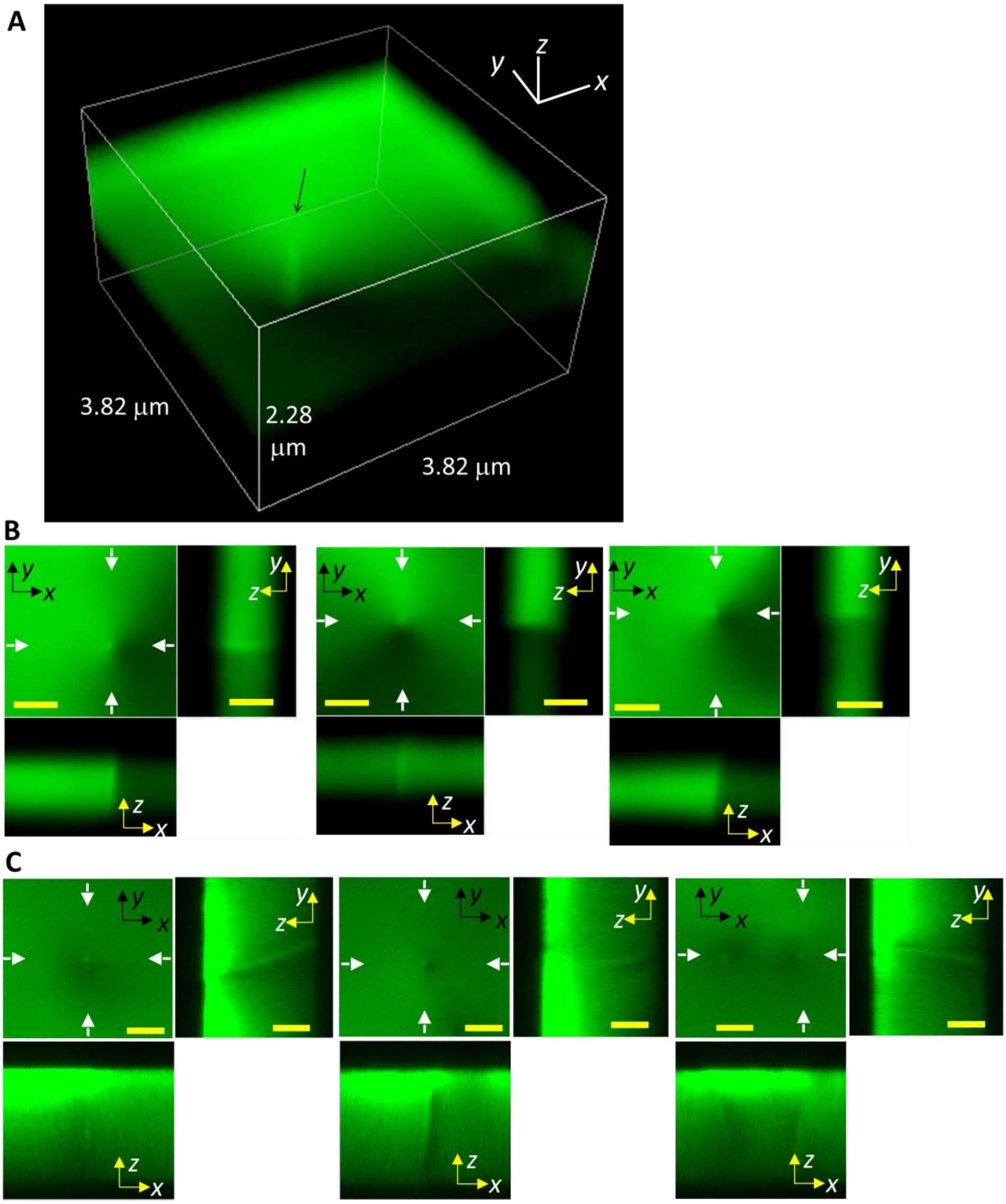

Supplementary Figure 4. Gleaming zigzag defects with *m* = ±1/2. In System-(ii), the top of the crest part was occasionally covered by the NLC, in which we had a chance to observe zigzag defect lines with $m = -1/2$ formed along the crests. (A) A schematic illustration of the cross section of the system (see also ref. 22). The position of zigzag defects with $m = \pm 1/2$ was observed using (B) POM with a sensitive tint (λ plate and (*C*-*E*) FOM. The zigzag lines in (C) with large and small periodicities correspond to defects with $m = +1/2$ and $-1/2$, respectively. The contrast of the intensity was enhanced to clearly show the gleaming zigzag defects with $m = -1/2$ in (D, E). (Scale bar: 20 μm.)

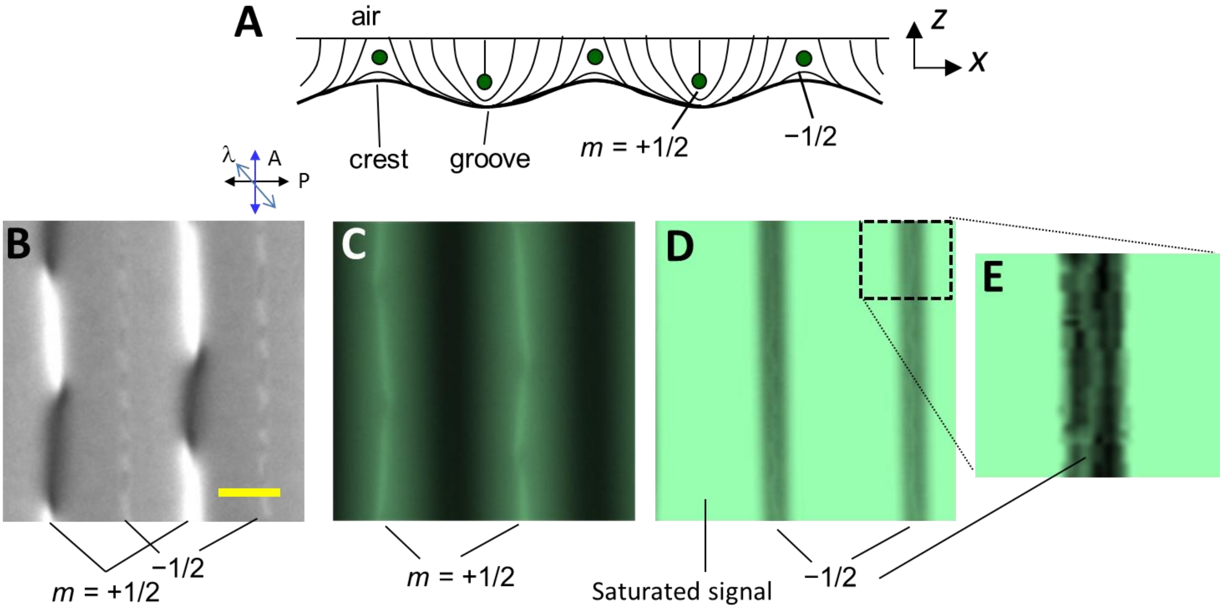

Supplementary Figure 5| Zigzag defects in a NLC of System-(ii). (Top) Typical POM images with different configurations of the polariser (P) and analyser (A). (Bottom) Geometrical confinement and semi-hybrid anchoring conditions (indicated by blue double-headed arrows) for a liquid filament of an NLC on a wrinkle groove are shown. The director structures ***n*** are shown by blue cylinders. The nail symbols (T) indicate the projection of ***n*** onto the plane of the surface (the head of the nail comes out of the surface). The twist configuration under the defect line causes the optical rotation, which is observable as bright parts in the POM image (top-right) under the crossed polarisers.

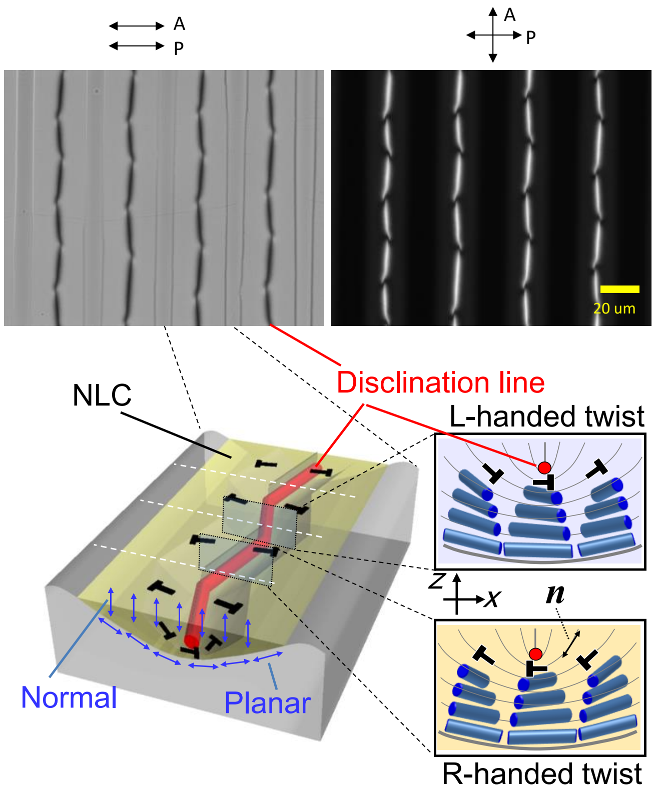

Supplementary Figure 6| Representative POM image of the wedge cell for the selection of observation region in System-(ii). Typical change of the colour from the edge to the thick part is shown. The yellow region indicated by an arrow is in the first order colour range of the Michel-Levy interference chart and suggests the thickness of approximately 2 ± 1 μm there. (Scale bar: 100 μm.)

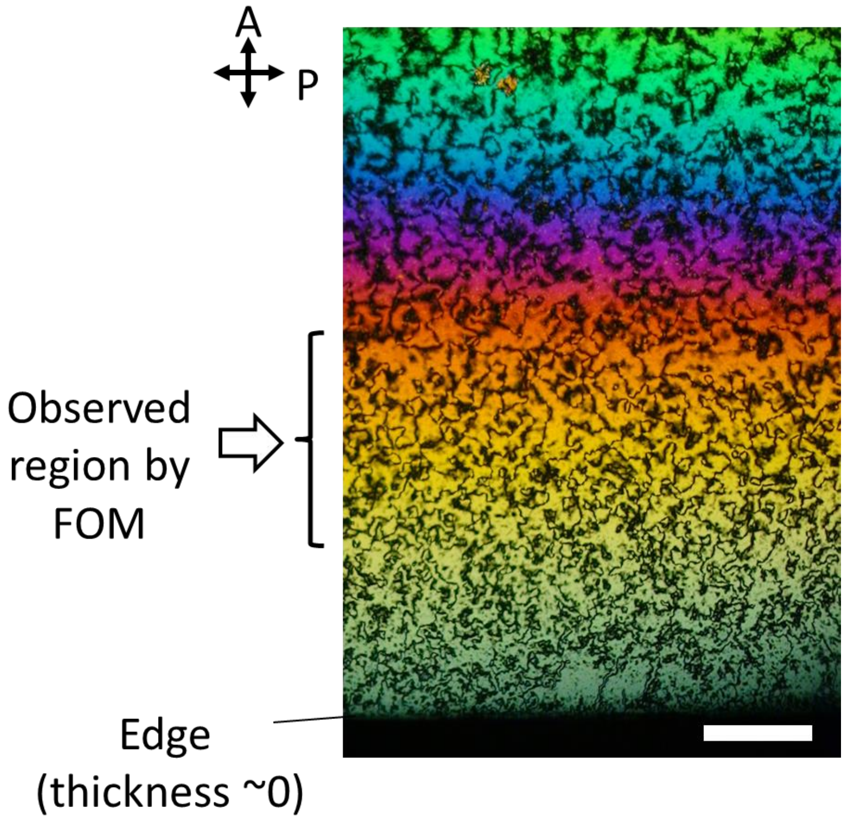

Supplementary Table 1| Other fluorescent LMW probes exhibiting gleaming defects. Depending on the fluorescent colour, different optical filter sets were used on FOM measurements, which are indicated as ‘blue’, ‘green’, and ‘red’ in the table (see Methods for the optical filter sets). Representative images observed in System-(ii) are shown as insets (Scale bars: 20 μm). Some of the probes, e.g., rubrene and fluorescein, show very weak emission and/or rapid optical degradation. A chiral probe, NBD-cholesterol, was also used, which induced right-handed twisted alignment. In System-(ii), the chiral dopant suppresses the formation of the zigzag from and induces a straight defect line[12,32] along the groove direction as shown in the middle inset.

Molecular name, abbreviation	MW (g/mol)	Concentration (wt%)	Emitted color (color filter on FOM)	Chirality
4-Nitro-7-piperazino-2,1,3-benzoxadiazole, NPB	249.23	~0.02	Green	No
Perylene, PL	252.32	~0.1	Blue/Green	No
2,2‘:5’,2’’:5’’,2’’’-α-quaterthiophene, QT	330.5	~0.1	Green	No
Fluorescein	332.31	~0.02	Green	No
Coumarin 504T, C504T	369.46	~0.1	Green	No
Coumarin 545T, C545T	430.57	~0.1	Green	No
Bis[2-(2-benzothiazolyl)phenolato]zinc(II), BPZ	517.93	~0.02	Blue/Green	No
Rubrene	532.69	~0.05	Green/Red	No
25-[N-[(7-nitro-2-1,3-benzoxadiazol-4-yl)methyl]amino]-27-norcholesterol, NBD-cholesterol	564.76	~0.5	Green	Yes, inducing right-handed twist in 5CB
N,N’-Bis(2,5-di-tert-butylphenyl)-3,4,9,10-perylenedicarboximide, BTBP	766.96	~0.02	Green	No
5CB (Host NLC)	249.36	-	-	No

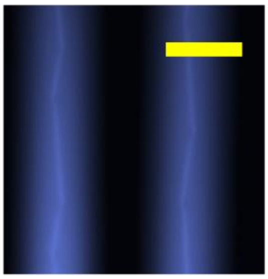

PL 0.1 wt%/5CB

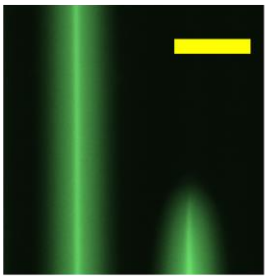

NBD-cholesterol 0.5 wt%/5CB

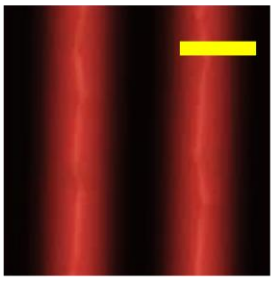

Rubrene 0.05 wt%/5CB
